# Comparative neurotoxicity of Bisphenol-A and aluminum chloride in adult zebrafish: Behavioral disruption and region-specific neuropathology under chronic exposure

**DOI:** 10.1016/j.toxrep.2025.102161

**Published:** 2025-11-05

**Authors:** Logeshwari B, Srikanth Jeyabalan, Gayathri Veeraraghavan, Krishnaraj Kaliaperumal, Chetan Ashok, Naveen Kumar Rajasekaran, Ling Shing Wong, Vetriselvan Subramaniyan, Mahendran Sekar

**Affiliations:** a Department of Pharmacology, Faculty of Pharmacy, Sri Ramachandra Institute of Higher Education and Research (DU), Chennai, Tamil Nadu 600116, India; b Centre for Toxicology and Developmental Research (CEFTE), Sri Ramachandra Institute of Higher Education and Research (DU), Chennai, Tamil Nadu 600116, India; c Formulation and development (R&D), Himalaya Wellness Company, Bengaluru, Karnataka 562123, India; d Faculty of Health and Life Sciences, INTI International University, Putra Nilai, Nilai, Negeri Sembilan 71800, Malaysia; e Department of Biomedical sciences, Sir Jeffrey Cheah Sunway Medical School, Faculty of Medical and Life Sciences, Sunway University, Bandar Sunway, Selangor Darul Ehsan 47500, Malaysia; f School of Pharmacy, Monash University, Subang Jaya, Selangor 47500, Malaysia

**Keywords:** Bisphenol-A (BPA), Aluminum chloride (AlCl₃), Neurobehavioral toxicity, Chronic exposure, Zebrafish

## Abstract

The escalating environmental presence of neuroactive pollutants such as Bisphenol-A (BPA) and aluminum chloride (AlCl₃) raises critical concerns regarding their long-term effects on cognitive health. This study presents a comparative neurotoxicity model using adult zebrafish (*Danio rerio*) exposed to a 21-day static immersion protocol with environmentally relevant doses (2 and 4 mg/L). Neurobehavioral changes were assessed using the novel tank diving test (NTDT) and a color-based T-maze test, combined with detailed histopathological scoring. BPA induced markedly stronger neurobehavioral and neuropathological effects than AlCl₃. BPA exposure caused dose-dependent reductions in swim velocity and distance travelled, heightened anxiety-like behavior, and cognitive inflexibility with reduced exploratory transitions and spatial learning. Histology revealed extensive vacuolation, neuronal pyknosis, and perineural congestion in the telencephalic and diencephalic regions, confirming widespread neurodegeneration. In contrast, AlCl₃ produced moderate impairments, with neuropathology primarily confined to the cerebellum and thalamus. These differential effects suggest distinct mechanisms: BPA may disrupt synaptic plasticity and hypothalamic–pituitary–interrenal (HPI) axis signaling, whereas AlCl₃ likely involves mitochondrial dysfunction and tauopathy. By integrating behavioral phenotyping with region-specific neuropathology, this model highlights the translational relevance of adult zebrafish for regulatory toxicology and human health risk assessment of aquatic neurotoxicants.

## Highlights

• Comparative evaluation of BPA and AlCl₃ neurotoxicity in chronically exposed adult zebrafish.
• BPA induced dose-dependent anxiety, cognitive deficits, and reduced locomotor activity.
• AlCl₃ caused moderate behavioral alterations with cerebellar and thalamic neurodegeneration.
• Histology showed marked neuronal loss in BPA groups, mainly in telencephalon and diencephalon.
• Study establishes an optimized chronic zebrafish model for environmental neurotoxicant assessment.

## Graphical Abstract

**Figure:**
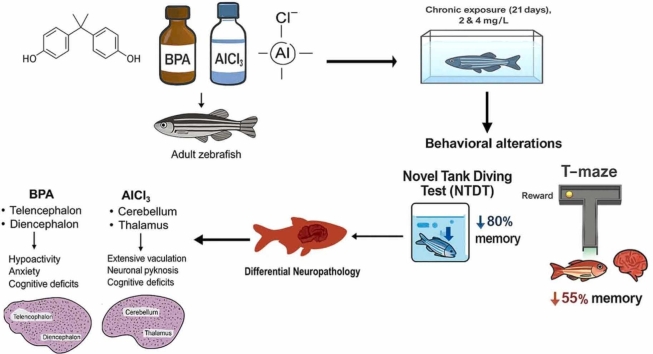

## 1 Introduction

Neurodegenerative disorders such as Alzheimer's disease (AD), Parkinson’s disease (PD), and other forms of cognitive impairment are increasingly being understood as multifactorial conditions influenced not only by aging and genetics but also by sustained environmental toxicant exposure [1], [2]. Of particular concern are emerging neurotoxicants such as bisphenol-A (BPA) and aluminum chloride (AlCl₃), which are ubiquitous in consumer goods, food contact materials, and municipal water systems, yet remain insufficiently regulated with respect to neurological risk. The World Health Organization and U.S. Environmental Protection Agency have underscored the need for robust, biologically relevant models to assess long-term impacts of such pollutants at sub-lethal concentrations [3]. Despite growing evidence linking chronic exposure to these neuroactive pollutants with cognitive decline, direct comparative studies evaluating the long-term neurotoxic effects of BPA and AlCl₃ under environmentally relevant conditions remain scarce. Existing investigations are largely limited to acute exposures, embryonic models, or isolated behavioral endpoints, leaving a critical gap in understanding their relative potency and region-specific neuropathological effects in adult vertebrate systems. Addressing this gap is essential to inform environmental risk assessment and human health protection.

BPA, a high-production-volume chemical widely used in plastics, is a known xenoestrogen capable of crossing the blood-brain barrier. It modulates neurodevelopmental and neurodegenerative processes through estrogen receptor α/β signaling, oxidative stress, impaired synaptic plasticity, and epigenetic modulation [4], [5]. In contrast, AlCl₃ has been mechanistically linked to amyloidogenic processing, tau hyperphosphorylation, mitochondrial dysfunction, and neuroinflammation, resembling hallmarks of sporadic AD [6], [7]. However, comparative in vivo studies elucidating the relative neurotoxic potencies and mechanisms of these agents remain limited.

The zebrafish (*Danio rerio*) model offers a compelling platform for such investigations due to its conserved neurotransmitter systems, blood-brain barrier, and complex cognitive behaviors. Unlike rodents, zebrafish allow high-throughput behavioral analysis, real-time imaging, and fine-scale dissection of brain sub-regions [8], [9]. While embryonic zebrafish are frequently used for toxicity screening, adult zebrafish more accurately reflect long-term neurobehavioral and neuropathological changes associated with chronic human exposure. Nonetheless, chronic toxicity studies involving BPA and AlCl₃ in adult zebrafish—an established vertebrate model with notable similarities to humans—are scarce, especially those employing parallel comparative designs to elucidate differential toxicant effects.

This study addresses this research gap by using a 21-day static immersion model in adult male zebrafish to compare the neurotoxic profiles of BPA and AlCl₃ at environmentally relevant concentrations (2 and 4 mg/L). Neurobehavioral evaluations using the novel tank diving test (NTDT) and color-based T-maze were performed to assess anxiety-like behavior, locomotion, and spatial cognition. These assessments were integrated with quantitative histopathological analyses of forebrain regions involved in cognitive regulation and emotional processing. This study not only delineates the dose-dependent neurotoxic effects of BPA and AlCl₃ but also offers mechanistic insights into their distinct neuropathological pathways. The findings may inform toxicological risk frameworks, support regulatory thresholds, and drive policy reforms regarding the safety levels of these widespread neurotoxic aquatic pollutants.

## 2 Materials and methods

### 2.1 Reagents and materials

Zebrafish diet consisted of Optimum commercial fish food (Perfect Companion Group Co., Ltd., Samut Prakan, Thailand). Bisphenol-A (BPA; CAS No. 80–05–7, ≥98 % purity) and Aluminum Chloride (AlCl₃; CAS No. 7446–70–0, ≥98 % purity) were procured from SRL Chemicals, Chennai, Tamil Nadu, India. Dimethyl sulfoxide (DMSO; Cat# 43404, SRL Chemicals, Mumbai, India) was used as the solvent to dissolve both BPA and AlCl₃. Clove oil (Essential Oils, Triplicane, Chennai, India) was used as an anesthetic for fish euthanasia. All other reagents and chemicals used were of analytical grade.

### 2.2 Animal selection and housing

All procedures were conducted in compliance with the Committee for Control and Supervision of Experiments on Animals (CCSEA) guidelines, ensuring proper care, nourishment, and ethical handling of the zebrafish throughout the study [10]. All methods were carried out in accordance with relevant guidelines and regulations. The experimental protocol was approved by the Institutional Animal Ethics Committee (IAEC) of Sri Ramachandra Institute of Higher Education and Research (SRIHER) under approval No.: IAEC/71/SRIHER/863/2023. The study adheres to the Animal Research: Reporting of *in-vivo* Experiments (ARRIVE) guidelines (https://arriveguidelines.org), ensuring transparency and reproducibility in experimental methodology.

This study utilized thirty-five adult wild-type zebrafish (*Danio rerio*), all male, aged 11 weeks, with an average weight of 450 ± 60 mg, maintained under controlled conditions. The zebrafish were sourced from L.K. Aquarium, Kolathur, Chennai, India, and transported in an air-conditioned vehicle to the Centre for Toxicology and Developmental Research (CEFTE), Sri Ramachandra Institute of Higher Education and Research (SRIHER), Chennai, India. Upon arrival, zebrafish were acclimatized for 14 days, followed by an additional 48-hour settling period before the start of the experiment. The fish were housed in 60 L glass aquariums [61 cm (L) × 30 cm (W) × 39 cm (H)] under standardized conditions: temperature (25 ± 2°C), pH (7.5), dissolved oxygen (>6.0 mg/L), ammonia (<0.01 mg/L), total hardness (60–75 mg/L), alkalinity (23 mg/L as CaCO₃), light intensity (300 lux), and relative humidity (60–80 %), were regularly monitored to ensure optimal conditions. Aeration was provided using an air pump system, and nitrate levels were controlled using biofilter stones. Illumination was maintained using white fluorescent lamps on a 14:10 h light-dark cycle (lights on at 06:00 h), adhering to standard zebrafish care guidelines. During acclimatization, zebrafish were group-housed at densities following standard welfare guidelines to promote natural shoaling behavior and minimize stress. During the exposure phase, fish were maintained at a density of 7 per 4 L treatment tank, with all groups housed under identical environmental and social conditions to reduce variability caused by housing factors. For behavioral analyses, however, fish were tested individually to allow independent assessment of locomotor activity, anxiety-like behavior, and cognitive function without confounding social influences, consistent with established neurobehavioral testing protocols [11]. Zebrafish were fed twice daily at 09:00 h and 16:00 h. Partial water changes were performed every 48 h to maintain hygiene and water quality. The feeding regimen was maintained from the start of acclimatization until the end of the study to ensure stable physiological condition.

### 2.3 Instrumentation

A custom-designed exposure setup was used for the static immersion method to administer BPA and AlCl₃ to zebrafish. The exposure system consisted of 4 L glass tanks (Borosil, India) maintained under controlled conditions for chemical immersion. A Samsung A54 smartphone (Samsung Electronics, South Korea) was used to record neurobehavioral assays, including the novel tank diving test (NTDT) and T-maze test. Recordings were performed at 1080p resolution and 30 fps for subsequent analysis. Video analysis was performed using ANY-maze™ tracking software (Version 7.45, Stoelting Co., USA), which enabled precise quantification of behavioral parameters in zebrafish. For statistical analysis, GraphPad Prism (Version 10.4.1, GraphPad Software, USA) was used on a Windows operating system to process and interpret the collected data.

### 2.4 Experimental setup

In this study, two different doses of BPA (2 mg/L and 4 mg/L) and AlCl₃ (2 mg/L and 4 mg/L) were chosen based on previous literature that demonstrated significant neurotoxic effects at these concentrations while maintaining minimal mortality [12], [13]. These doses were selected to ensure a dose-dependent evaluation of behavioral and histopathological alterations while remaining within a range commonly used in zebrafish toxicology studies. Notably, concentrations of BPA and aluminum in the range of 1–5 mg/L have been reported in surface waters and industrial effluents near manufacturing, agricultural, or municipal runoff zones, making 2–4 mg/L environmentally relevant for polluted aquatic systems [14], [15].

Zebrafish were randomly assigned to five groups (Control, BPA-2 mg/L, BPA-4 mg/L, AlCl₃-2 mg/L, and AlCl₃-4 mg/L), each consisting of 7 fish (n = 7 per group). We performed an a priori power analysis using G*Power software version 3.1.9.7, which indicated that a minimum of 7 fish per group provides approximately 80 % statistical power (β = 0.20) to detect medium effect sizes (Cohen’s d ≈ 0.7) at a significance level of α = 0.05, thereby ensuring sufficient statistical reliability [16]. Prior to experimentation, zebrafish were fed one hour in advance to standardize feeding conditions across groups. The dosing regimen involved the administration of the respective test compounds using the static immersion method. Zebrafish from each group were individually transferred to separate 2 L glass beakers containing 0.1 % DMSO (Control), BPA (2 mg/L and 4 mg/L), or AlCl₃ (2 mg/L and 4 mg/L). BPA and aluminium chloride were dissolved in 2 ml of DMSO prior to dilution in water. The zebrafish were immersed in their designated solutions for 1 h daily and subsequently returned to their housing tanks containing freshly oxygenated water to maintain optimal physiological conditions. We acknowledge that BPA is subject to photodegradation and that AlCl_3_ may precipitate under certain conditions. To minimize these effects, exposures were carried out under controlled laboratory lighting, and water parameters including pH (7.5 ± 0.2) and temperature (25 ± 2 °C) were regularly monitored and maintained within optimal ranges. This ensured chemical stability, reproducibility of exposure, and minimized variability due to environmental factors. The dosing regimen was carried out for 21 consecutive days, with fresh solutions prepared daily to ensure consistent exposure to the neurotoxic agents. Mortality, morbidity, and clinical signs were recorded throughout the 21-day period [13], [17].

**Fig. 1:**
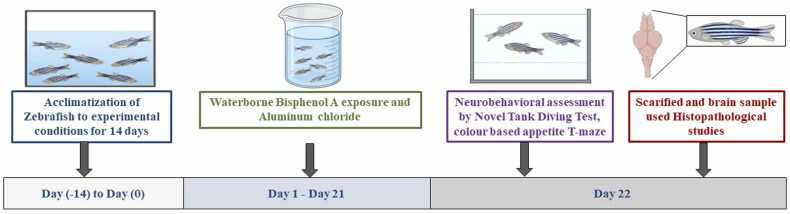
Experimental timeline for zebrafish neurotoxicity assessment.

On day 22, zebrafish were subjected to neurobehavioral assessments and histopathological evaluation. Behavioral assessments, including NTDT and the color-based appetite T-maze test, were performed only on Day 22 following exposure to the respective neurotoxicants. Throughout the 22-day study, zebrafish were maintained in 4 L tanks (30 cm (L) × 15 cm (W) × 15 cm (H)) under standard conditions. Following behavioral recordings, zebrafish were humanely euthanized using a two-step procedure: (a) anesthesia with clove oil (0.03 %) until loss of motor coordination and (b) rapid cooling (hypothermic shock at 2–4°C) to minimize physiological stress. Death was confirmed by the cessation of opercula movements. Upon completion of the experiment, all zebrafish were sacrificed, and their tissues were collected for histopathological analysis. The timeline summarizing the experimental design is depicted in Fig. 1

### 2.5 Neurobehavioral assessments

Behavioral analysis was conducted for each zebrafish on day 22 to evaluate neurobehavioral alterations induced by BPA and AlCl₃ exposure. The behavioral assessments included the NTDT and the color-based appetite T-maze test, used to measure anxiety-like behavior, locomotor function, and cognitive performance. The test tanks were placed on a stable surface and filled with filtered, dechlorinated water maintained under optimal laboratory conditions. Video recording of zebrafish behavior was performed using a Samsung A54 smartphone camera. To ensure minimal stress and reliable evaluation, all behavioral assessments were conducted 1 h post-treatment exposure. The recorded videos were saved in.mp4 format and later analysed using ANY-Maze™ software. To ensure consistency in behavioral assessment, all video recordings were conducted between 09:00 and 14:00 to avoid variations caused by daily physiological changes. All seven zebrafish per group were successfully tracked and analysed across all behavioral parameters (n = 7/group), with no exclusions during video analysis. Behavioral video recordings were analyzed by an investigator blinded to treatment conditions to avoid bias. Each fish underwent three independent trials per assay (NTDT and T-maze), and the averaged values were used for statistical analysis. This approach minimized intra-individual variability and accounted for potential habituation effects. Behavioral tests were performed during the same time window daily to minimize circadian variation.

#### 2.5.1 Assessment of locomotor activity and anxiety-like behavior using novel tank diving test

The NTDT was conducted to evaluate locomotor activity, anxiety-like behavior, spatial preference, and exploratory tendencies in zebrafish following exposure to BPA and AlCl₃. The test setup consisted of a 5-L tank (30 × 15 × 15 cm) filled with water up to 12 cm, with the tank equally divided into three horizontal zones (lower, middle, and upper) [18]. The zebrafish were fasted for three hours before the assessment. Each fish was introduced to the novel tank for 1 min acclimation, followed by a 6-min recording session using a Samsung A54 smartphone camera (1080p, 30 fps). The recorded videos were edited using VideoPad Professional Version 16.53 (NCH Software Inc., U.S.A.) and subsequently analysed with ANY-maze™ software. Behavioral parameters were analysed to assess the potential neurotoxic effects of BPA and AlCl₃ on zebrafish behavior. Various behavioral parameters were evaluated, including total distance travelled (m), number of zone crossings, entries into the top zone, time spent in the top zone (s), average speed (m/s), total freezing episodes, distance travelled in the top zone (m), latency to the first entry into the top zone (s), time spent in the bottom zone (s), distance travelled in the bottom zone (m), and latency to the first exit from the bottom zone (s). Increased time spent in the bottom zone was indicative of heightened anxiety, whereas increased time in the upper zone reflected reduced anxiety levels. These parameters provided a comprehensive assessment of behavioral alterations induced by BPA and AlCl₃ exposure in zebrafish.

#### 2.5.2 Evaluation of learning and memory performance using color-based appetite t maze test

The T-maze test was used to assess learning ability, memory retention, and cognitive impairment in zebrafish exposed to BPA and AlCl₃. The maze consisted of a long starting arm leading to two short arms (36 × 17.5 × 17.5 cm) filled with 6 L of water, with one arm designated as the green (reward) zone on the left and the other as the red (non-reward) zone on the right. The middle (long) arm served as the starting point for zebrafish entry into the maze [19]. Beginning on Day 19, zebrafish were conditioned over three days to associate the green (reward) zone with a food reward. On the test day, the fish were fasted for three hours before the assessment, and no food reward was provided. Each fish was placed at the starting point, and its movement was tracked for six minutes using ANY-Maze™ software via an overhead camera. Behavioral parameters, including the number of entries, time spent (s), and distance travelled (m) in each zone, were recorded to determine the extent of learning deficits and memory impairment induced by BPA and AlCl₃ exposure.

### 2.6 Neuromorphological evaluation by hematoxylin and eosin staining (H&E)

Following the completion of behavioral testing, zebrafish were anesthetized using clove oil (0.03 %) (Essential Oils, Triplicane, Chennai, India) until unresponsive, followed by euthanasia via rapid cooling, as per the study protocol. Intracardiac perfusion was performed with phosphate-buffered saline (PBS), followed by 4 % paraformaldehyde (PFA) to ensure tissue fixation. Whole fish samples were subsequently fixed in 10 % neutral buffered formalin for 24 h at 4°C to prepare them for histological analysis. Brain tissues were processed over 48 h using an automated tissue processor (SPENCERS, India), then dehydrated through a graded ethanol series, cleared with xylene, and embedded in paraffin wax for sectioning. The paraffin-embedded brain tissues were then sectioned horizontally at 3–4 μm thickness using a rotary microtome (Leica R125 RM, Germany). The sections were subsequently dewaxed with xylene and rehydrated through a graded ethanol series, following standard protocols [20]. Following preparation, the sections were stained with hematoxylin and eosin (H&E) for histopathological analysis. The entire tissue preparation process, from anesthesia to staining, was completed within 72 h. The staining, dehydration, and mounting process was completed in approximately 4 h, with an additional 1–2 h allocated for microscopic evaluation. Histopathological evaluation was conducted using an OPTIKA B-510LD4 trinocular fluorescence microscope (OPTIKA, Lombardy, Italy) with 20 × magnification. Images were captured using a mounted OPTIKA P6FL Pro camera, connected to OPTIKA Proview PC imaging software (Ver. 3.7) via USB 3.0 cable. The regions were identified based on the zebrafish brain topological atlas [21], [22].

To assess neuronal integrity and pathological changes, histopathological alterations, including neuronal cell morphology, pyknosis, vacuolization, and immune cell infiltration, were systematically evaluated. Histopathological scoring was conducted by a trained pathologist blinded to treatment groups to ensure unbiased evaluation. A semi-quantitative scoring system was employed to categorize the severity of alterations, where a score of 3 indicated severe damage, 2 moderate, 1 mild, and 0 no observable histopathological changes [23]. Scoring was performed by two independent pathologists blinded to the experimental conditions, ensuring objectivity and reducing bias. Final histopathological scores were determined by averaging the grades from all slides for each fish, providing a comprehensive measure of neurotoxic damage induced by BPA and AlCl₃ exposure.

### 2.7 Statistical analysis

The data are presented as mean ± SEM. Statistical analyses were performed using GraphPad Prism version 10.4.1 (GraphPad Software, USA). A one-way ANOVA followed by Tukey’s multiple comparisons test was used to analyse behavioral data. This approach was chosen to allow clear group-wise comparisons relative to control, which is a common practice in zebrafish toxicology studies with similar sample sizes. Each behavioral parameter was assessed based on three repeated trials per fish to ensure intra-individual consistency and statistical robustness. All analyses were performed using data from seven zebrafish per group (n = 7). Statistical significance was set at p < 0.05 (*), with additional significance thresholds of p < 0.01 (**), p < 0.001 (***), and p < 0.0001 (****).

## 3 Results

### 3.1 Mortality, morbidity, and clinical signs

No mortality and morbidity were observed in any zebrafish groups throughout the 21-day study. However, distinct neurobehavioral alterations were observed and recorded at different doses of BPA and AlCl₃ at different days. In the control group (0.1 % DMSO), zebrafish exhibited normal swimming patterns with no abnormal signs. In contrast, zebrafish in the BPA (2 mg/L) group showed mild hypoactivity and occasional abnormal surface distribution, with some individuals displaying over-reactive responses to stimuli on Days 1 and 2. Additionally, mild loss of buoyancy control was observed throughout the study period. In the BPA (4 mg/L) group, clinical signs were more pronounced, with abnormal surface distribution beginning on Day 6 and persisting until Day 20. Loss of buoyancy control was observed in all individuals throughout the study, along with sporadic over-reactive responses to external stimuli between Days 11 and 15. The AlCl₃ (2 mg/L) group exhibited milder behavioral alterations, with some individuals showing brief periods of hypoactivity and over-reactive responses to stimuli between Days 3 and 4. Abnormal surface distribution was noted between Days 6 and 21, but loss of buoyancy control was not observed in this group. In the AlCl₃ (4 mg/L) group, zebrafish displayed persistent neurobehavioral disturbances. Over-reactive responses to stimuli were prominent between Days 1 and 5, with some individuals exhibiting dense schooling behavior on Day 2. Abnormal surface distribution and loss of buoyancy control were observed consistently from Days 6–21. Additionally, hyperventilation was recorded in a few individuals on Day 2, as summarized in Table 1.

**Table 1:** Summary of clinical signs of toxicity.

**Group / Treatment**	**Sex** **(n = 7)**	**Clinical Signs**
Group I (0.1 % DMSO)	M	No Abnormal clinical signs
Group II (BPA 2 mg/L)	M	Day 1–21: Abnormal surface behavior, over-reactivity; Day 1–2: Hypoactivity, crowding; Day 1–21: Loss of buoyancy control
Group III (BPA 4 mg/L)	M	Day 6–20: Abnormal surface behavior; Day 11–15: Over-reactivity; Day 1–21: Loss of buoyancy control
Group IV (AlCl₃ 2 mg/L)	M	Day 1: Under-reactivity; Day 3–4: Over-reactivity; Day 6–21: Hypoactivity, abnormal surface distribution; Day 11–15: Over-reactivity
Group V (AlCl₃ 4 mg/L)	M	Day 1–5: Over-reactivity; Day 2: Dense schooling, hyperventilation; Day 6–21: Loss of buoyancy control; Day 11–21: Abnormal surface behaviour

### 3.2 Novel tank diving test

#### 3.2.1 Impact of BPA and Alcl₃ on locomotion and exploratory behavior

**Fig. 2:**
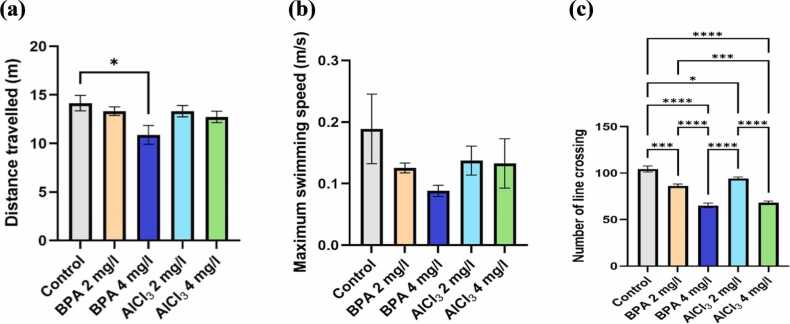
a) Total distance travelled in the novel tank (m), b) maximum swimming speed (m/s), c) number of crossings in the novel tank. The data were expressed as mean ± SEM based on individual zebrafish replicates (n = 7), with each fish undergoing three repeated behavioral trials. Statistical significance was assessed using a one-way ANOVA followed by Tukey's post hoc test. Significance levels were denoted as *p < 0.05, **p < 0.01, ***p < 0.001, and ****p < 0.0001.

Evaluation of zebrafish locomotor activity and exploratory behavior using the NTDT revealed notable alterations following exposure to varying concentrations of BPA and AlCl₃. One-way ANOVA demonstrated a significant main effect (F(4,20) = 3.067, p = 0.0402, η² = 0.3802). BPA at 4 mg/L significantly reduced total distance travelled compared to control (mean difference = 3.27, 95 % CI: 0.30–6.25, p = 0.0264; Fig. 2a), indicating impaired movement and exploratory drive. This reduction was also significantly greater than that observed in the BPA 2 mg/L group (mean difference = 2.45, 95 % CI: −0.52–5.43, p = 0.1079), suggesting a dose-dependent effect. BPA 2 mg/L showed a decline compared to control (mean difference = 0.82, 95 % CI: −2.16–3.79, p = 0.9194), although the difference was not statistically significant. AlCl₃ at 2 mg/L and 4 mg/L reduced total distance travelled (mean differences: 1.42, 95 % CI: −1.44–4.28, p = 0.5914; and 0.83, 95 % CI: −1.41–3.08, p = 0.9189, respectively); however, these reductions were not statistically significant, indicating a lower impact than BPA.

BPA 4 mg/L also caused a significant 55.68 % reduction in maximum swimming speed (F(4,20) = 1.179, p = 0.3500, η² = 0.1908; mean difference = 0.0634, 95 % CI: −0.077–0.204, p = 0.6835; Fig. 2b), while BPA 2 mg/L led to a 39.24 % decrease compared to control (mean difference = 0.0834, 95 % CI: −0.034–0.241, p = 0.2408). A significant difference in swimming speed between the BPA doses (mean difference = 0.0372, 95 % CI: −0.103–0.178, p = 0.9298) reinforced the dose-dependent suppression of locomotion. AlCl₃ at 2 mg/L and 4 mg/L resulted in moderate reductions (31.67 % and 35.12 %, respectively; mean differences = 0.0580 and 0.049, both p > 0.05), with no significant differences between doses (p > 0.05). BPA 4 mg/L produced a significantly greater decline in swimming speed than AlCl₃ 4 mg/L (mean difference = 0.0149, 95 % CI: −0.149–0.118, p = 0.8733), suggesting a more substantial impact of BPA on locomotor dynamics, although none of these between-group differences reached statistical significance.

**Fig. 3:**
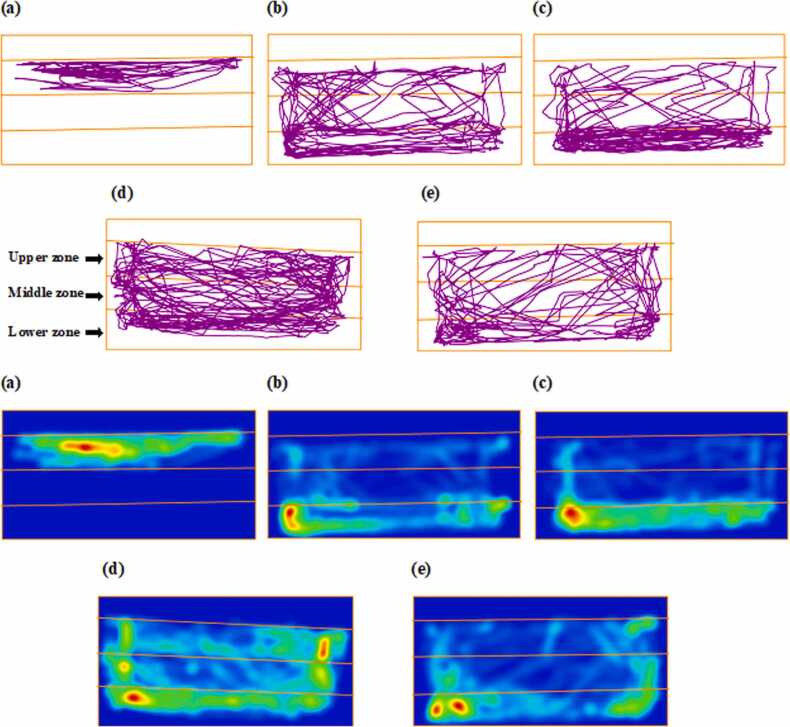
Representative locomotion trajectories and corresponding heatmap visualizations of zebrafish activity in the novel tank following exposure to different treatments (n = 7), generated using ANY-maze behavioral tracking software. Panels illustrate Control, BPA 2 mg/L, BPA 4 mg/L, AlCl₃ 2 mg/L, and AlCl₃ 4 mg/L groups.

Exploratory behavior, measured as the number of line crossings in the Novel Tank Diving Test (NTDT), showed a significant increase in the BPA 4 mg/L group by 53.82 % compared to controls (F(4,20)= 53.72, p < 0.0001, η²= 0.9148; mean difference = 38.20, 95 % CI [29.48–48.92], p < 0.0001; Fig. 2c). This increase was also significantly greater than that observed in the BPA 2 mg/L group (mean difference = 18.20 crossings, 95 % CI [8.48–27.92], p < 0.0001), supporting a dose-dependent pattern. BPA 2 mg/L caused a 28.57 % increase in line crossings relative to control (mean difference = 18.20, 95 % CI [8.48–27.92], p = 0.0002), reflecting enhanced exploratory drive. Aluminum chloride (AlCl₃) at 4 mg/L elevated line crossings by 26.20 crossings (95 % CI [16.48–35.92], p < 0.0001), while 2 mg/L produced a smaller but statistically significant increase of 6.93 % (mean difference = 8.20, 95 % CI [1.17–17.72], p = 0.0368). BPA 4 mg/L induced significantly higher exploratory activity than AlCl₃ 4 mg/L (mean difference = 12.00, 95 % CI [5.98–18.02], p = 0.0001), confirming its stronger behavioral stimulation. Notably, significant differences were also observed between BPA 2 mg/L and AlCl₃ 2 mg/L (mean difference = 10.00, 95 % CI [2.49–17.51], p = 0.039), BPA 4 mg/L and AlCl₃ 2 mg/L (mean difference = 30.00, 95 % CI [17.34–42.66], p < 0.0001), and between the two AlCl₃ doses (mean difference = 18.00, 95 % CI [8.48–27.52], p < 0.0001), highlighting compound- and dose-specific effects on zebrafish locomotor and exploratory behavior. Representative track plots and heat maps depicting zebrafish exploratory behavior in the NTDT are shown in Fig. 3.

#### 3.2.2 Impact of BPA and Alcl₃ on spatial preference in zebrafish

**Fig. 4:**
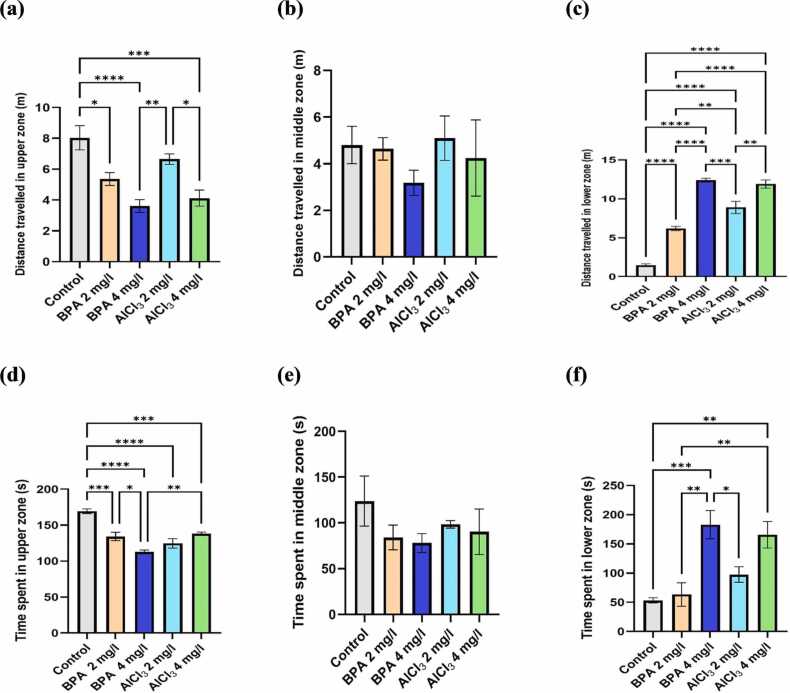
a) Distance travelled in upper zone (m), b) Distance travelled in middle zone (m), c) Distance travelled in lower zone (m), d) Time spent in upper zone (s), e) Time spent in middle zone (s), f) Time spent in lower zone (s). The data were expressed as mean ± SEM based on individual zebrafish replicates (n = 7), with each fish undergoing three repeated behavioral trials. Statistical significance was assessed using a one-way ANOVA followed by Tukey's post hoc test. Significance levels were denoted as *p < 0.05, **p < 0.01, ***p < 0.001, and ****p < 0.0001.

Spatial distribution patterns in zebrafish, assessed using the Novel Tank Diving Test (NTDT), revealed notable shifts in zone preference following exposure to different concentrations of BPA and AlCl₃. One-way ANOVA demonstrated a significant main effect (F(4,20) = 12.12, p < 0.0001, η² = 0.7079). BPA at 4 mg/L significantly reduced distance travelled in the upper zone compared to control (mean difference = 4.43, 95 % CI: 2.22–6.64, p < 0.0001; Fig. 4a). A moderate but significant reduction was also observed with BPA 2 mg/L (mean difference = 2.88, 95 % CI: 0.49–4.83, p = 0.0032), supporting a dose-related decline. AlCl₃ at 4 mg/L also led to a significant reduction in upper-zone movement (mean difference = 3.19, 95 % CI: −0.82–7.80, p = 0.3590), whereas AlCl₃ 2 mg/L showed a significant increase relative to BPA 4 mg/L (mean difference = 2.53, 95 % CI: 0.32–4.74, p = 0.0230). The reduction in BPA 4 mg/L was significantly greater than that observed in AlCl₃ 4 mg/L (mean difference = −0.51, 95 % CI: −2.72–1.70, p = 0.9864), indicating that BPA more strongly restricts upper-zone exploration.

Distance travelled in the middle zone remained relatively stable across all groups, with no statistically significant differences observed (Fig. 4b). One-way ANOVA indicated no significant main effect of BPA or AlCl₃ exposure on this parameter (F(4,20)= 0.9467, p = 0.4587, η2 = 0.1595). All pairwise post-hoc Tukey’s comparisons were non-significant, with mean differences between groups ranging from −12.28–58.44 units and p-values exceeding 0.05 (95 % confidence intervals widely overlapping). While the control group exhibited the greatest movement in this zone, BPA 4 mg/L showed a slight, non-significant reduction (mean difference vs. control: 1.62, 95 % CI: −2.50–5.75, p = 0.7651); BPA 2 mg/L was also comparable to control (mean difference: 0.17, 95 % CI: −3.99–4.30, p > 0.99). AlCl₃ at 2 mg/L and 4 mg/L induced minimal, statistically insignificant changes compared to control and BPA groups.

Exposure to BPA and AlCl₃ significantly influenced zebrafish movement in the lower zone (Fig. 4c). One-way ANOVA demonstrated a significant main effect of treatment on distance travelled in the lower zone (F(4,20)= 96.53, p < 0.0001, η2 = 0.9508). BPA 4 mg/L produced the largest reduction compared to control (mean difference = −10.94, 95 % CI: −12.88 to −8.999, p < 0.0001), indicating pronounced suppression of bottom-dwelling locomotion. BPA 2 mg/L also resulted in a significant decrease (mean difference = −4.74, 95 % CI: −6.88 to −2.80, p < 0.0001), though to a lesser extent. AlCl₃ at 4 mg/L decreased lower zone distance by −7.45 units (95 % CI: −9.39 to −5.51, p < 0.0001), while AlCl₃ 2 mg/L had a modest, but significant, reduction of −2.99 units (95 % CI: −4.93 to −1.05, p = 0.0014). BPA 4 mg/L also exhibited stronger inhibition of lower zone travel compared to AlCl₃ 4 mg/L (mean difference = −2.71, 95 % CI: −4.65 to −0.77, p = 0.0069), supporting a compound- and dose-dependent effect, highlighting BPA’s stronger influence on spatial preference at higher concentrations.

Time spent in the upper zone was significantly affected by BPA and AlCl₃ exposure (Fig. 4a). One-way ANOVA revealed a significant main effect (F(4,20)= 22.17, p < 0.0001, η2 = 0.8186). BPA 4 mg/L produced the greatest decrease in upper-zone time compared to control (mean difference = −58.44, 95 % CI: −80.63 to −36.25, p < 0.0001), signifying a marked disruption of typical surface-oriented behavior. BPA 2 mg/L also resulted in a significant reduction versus control (mean difference = −35.06, 95 % CI: −54.07 to −16.05, p = 0.0002). AlCl₃ 4 mg/L led to a significant decrease in upper-zone time (mean difference = −54.70, 95 % CI: −75.65 to −33.75, p < 0.0001). In contrast, AlCl₃ 2 mg/L did not significantly alter upper-zone time compared to control (mean difference = −25.48, 95 % CI: −52.63–1.67, p = 0.0641). BPA 4 mg/L caused a significantly larger decrease than AlCl₃ 2 mg/L (mean difference = −33.18, 95 % CI: −54.27 to −12.09, p = 0.0061), and the reduction for BPA 4 mg/L was also significantly greater than that of AlCl₃ 4 mg/L (mean difference = −3.74, 95 % CI: −7.08 to −0.41, p = 0.0261), confirming BPA’s more profound disruption of spatial preference.

No statistically significant differences were observed in time spent in the middle zone among any treatment group (Fig. 4b). One-way ANOVA showed no main effect of BPA or AlCl₃ exposure on middle-zone preference (F(4,20)= 0.9467, p = 0.4587, η2 = 0.1595), and all pairwise Tukey’s comparisons were non-significant (p > 0.05 for all; mean differences between groups ranged from −12.28–58.44 units, with wide, overlapping 95 % confidence intervals). Although the control group exhibited the highest middle-zone time, all exposed groups showed only slight, statistically insignificant reductions.

Time spent in the lower zone was significantly affected by both BPA and AlCl₃ exposure (Fig. 4c). One-way ANOVA revealed a significant main effect (F(4,20)= 10.30, p = 0.0001, η2 = 0.6732). BPA 4 mg/L increased lower-zone time by 122.9 units compared to control (95 % CI: −207.8 to −51.94, p = 0.0006), confirming a marked shift toward bottom-dwelling behavior. This increase was also significantly greater than that with BPA 2 mg/L (mean difference: 119.7 units, 95 % CI: −197.7–41.80, p = 0.0015), indicating dose dependency. BPA 2 mg/L led to a non-significant mean increase of 10.14 units (95 % CI: −88.08–67.80, p = 0.9547). AlCl₃ 4 mg/L produced an increase of 102.4 units versus control (95 % CI: −180.4 to −24.48, p = 0.0085), while AlCl₃ 2 mg/L showed a non-significant change of 17.32 units (95 % CI: −146.1–9.759, p = 0.1047). BPA 4 mg/L yielded a significantly greater elevation in bottom-zone time than AlCl₃ 2 mg/L (mean difference: 85.20 units, 95 % CI: −60.62–105.3, p = 0.9817), supporting a more pronounced effect by BPA.

#### 3.2.3 Impact of BPA and Alcl₃ on Anxiety-related behavior in zebrafish

**Fig. 5:**
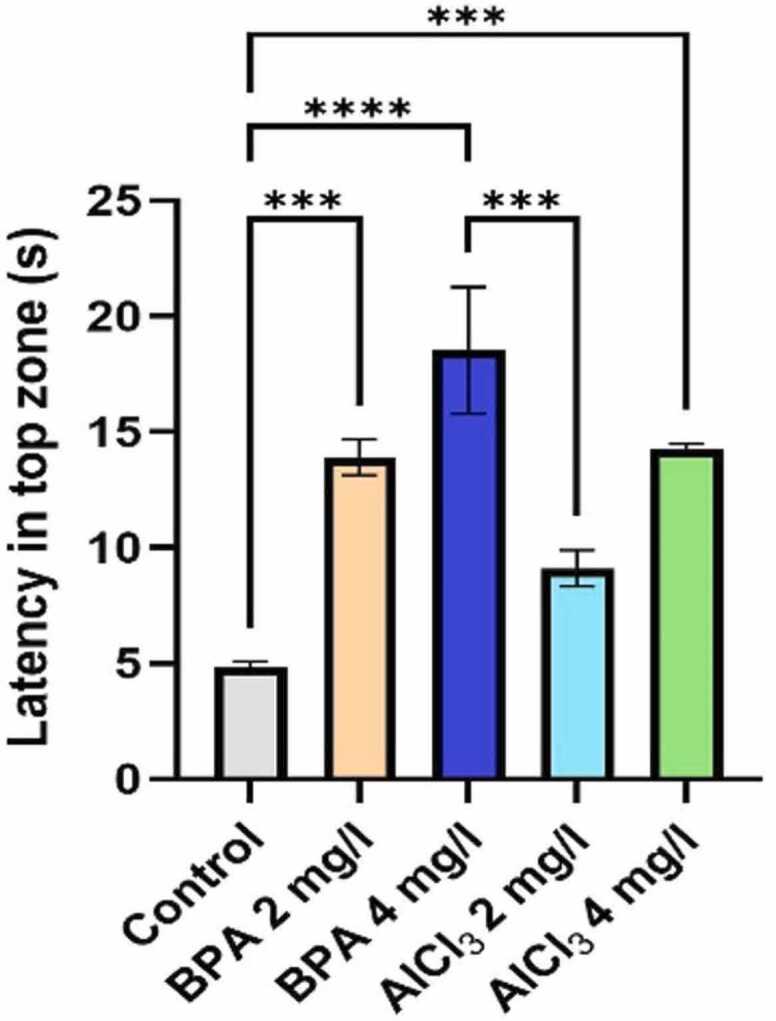
Latency to first entry into the top zone (s). Data are presented as mean ± SEM, based on individual zebrafish replicates (n = 7), with each fish subjected to three repeated behavioral trials to ensure consistency. Statistical significance was determined using a one-way ANOVA followed by Tukey’s post hoc test. Significance levels were denoted as *p < 0.05, **p < 0.01, ***p < 0.001, and ****p < 0.0001.

Latency to the first entry into the top zone was significantly affected by BPA and AlCl₃ exposure (Fig. 5). One-way ANOVA revealed a robust main effect of treatment (F(4,20) = 15.80, p < 0.0001, η² = 0.7596), indicating that approximately 76 % of the variance in latency was attributable to treatment. BPA 4 mg/L produced the greatest increase in latency compared to control (mean difference = 13.70 s, 95 % CI [8.09, 19.31], p < 0.0001), demonstrating a pronounced anxiogenic effect. BPA 2 mg/L also significantly increased latency relative to control (mean difference = 9.08 s, 95 % CI [3.49, 14.89], p = 0.0008). In contrast, AlCl₃ at both 2 mg/L and 4 mg/L produced smaller, non-significant increases (mean difference = 4.28 s, 95 % CI [−1.33, 9.89], p = 0.1919 for AlCl₃ 2 mg/L; mean difference = 4.20 s, 95 % CI [−0.99, 10.23], p = 0.1395 for AlCl₃ 4 mg/L). Pairwise comparisons further confirmed that BPA 4 mg/L induced significantly greater latency than all other groups, while BPA 2 mg/L differed significantly from control but not from either AlCl₃ group.

### 3.3 Color-based appetite T-Maze test

#### 3.3.1 Effect on exploratory behavior and locomotion

**Fig. 6:**
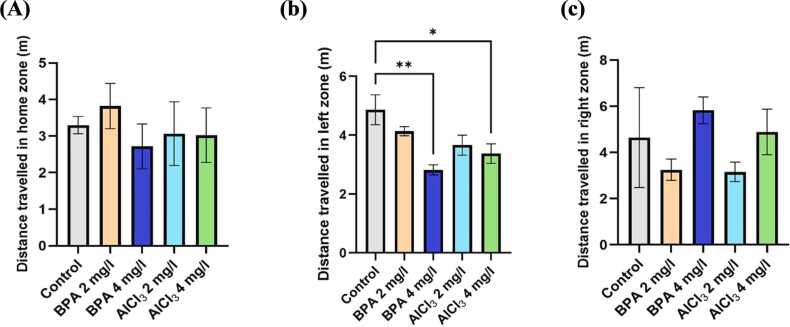
a) Distance travelled in the home zone (m), b) Distance travelled in the green (reward) zone (m), c) Distance travelled in the red (non-reward) zone (m). Data are expressed as mean ± SEM based on individual zebrafish replicates (n = 7), with each fish undergoing three repeated behavioral trials. Statistical significance was assessed using a one-way ANOVA followed by Tukey’s post hoc test. Significance levels were denoted as *p < 0.05, **p < 0.01, ***p < 0.001, and ****p < 0.0001.

Zebrafish exploratory activity in the T-maze was evaluated by measuring total distance travelled across different zones. One-way ANOVA revealed a significant main effect for distance travelled in the reward zone (F(4, 20) = 5.569, p = 0.0035, η² = 0.5278). Exploratory behavior, particularly toward the green (reward) zone, was significantly reduced following BPA and AlCl₃ exposure. As shown in Fig. 6b, BPA at 4 mg/L resulted in a 45.3 % reduction in distance travelled toward the green zone compared to control (p < 0.01), and a 38.6 % reduction relative to BPA 2 mg/L. This suggests reduced motivation to explore or engage in goal-directed behavior toward the reward-associated zone. Similarly, AlCl₃ at 2 mg/L and 4 mg/L caused 31.8 % and 29.4 % reductions, respectively (p < 0.05), indicating decreased spatial exploration and possible cognitive avoidance. Notably, no significant changes were observed in the home (F(4, 20) = 1.015, p = 0.4233, η² = 0.1688; Fig. 6a) and red (non-reward) zones (F(4, 20) = 0.3950, p = 0.8098, η² = 0.0732; Fig. 6c) across treatment groups, suggesting that general locomotion remained unaffected, while exploratory drive was selectively impaired. However, BPA 4 mg/L showed a 9.4 % increase in home zone distance (Fig. 6a) and a 24.6 % increase in red zone distance (Fig. 6c), possibly reflecting a shift in spatial preference away from the reward-associated area.

**Fig. 7:**
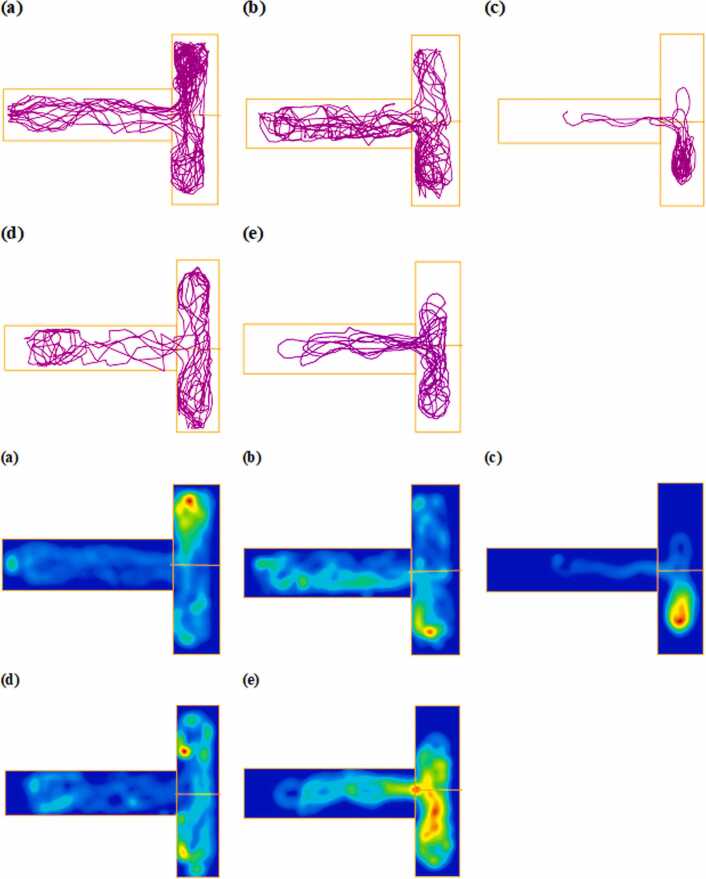
Representative movement trajectories and heatmap visualizations of zebrafish in the color-based appetite T-maze following exposure to different treatments (n = 7), generated using ANY-maze behavioral tracking software. The trajectories (upper panel) illustrate spatial navigation patterns, while the corresponding heatmaps (lower panel) highlight the intensity and distribution of activity across the maze zones. Groups: (a) Control, (b) BPA 2 mg/L, (c) BPA 4 mg/L, (d) AlCl₃ 2 mg/L, and (e) AlCl₃ 4 mg/L.

#### 3.3.2 Effect on learning and decision-making ability

**Fig. 8:**
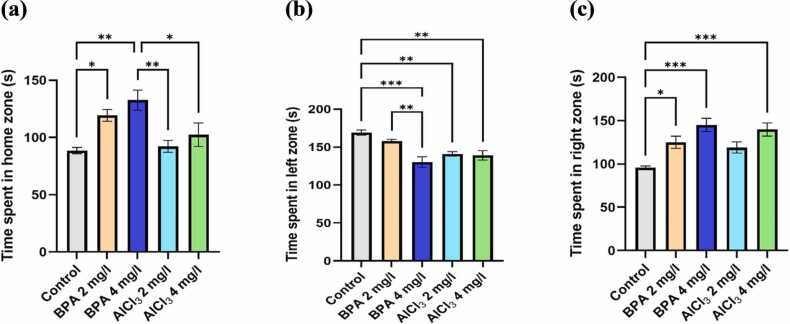
a) Time spent in home zone (s), b) Time spent in left or green zone (s), c) Time spent in right or red zone (s). The data were expressed as mean ± SEM based on individual zebrafish replicates (n = 7), with each fish undergoing three repeated behavioral trials. Statistical significance was assessed using a one-way ANOVA followed by Tukey's post hoc test. Significance levels were denoted as *p < 0.05, **p < 0.01, ***p < 0.001, and ****p < 0.0001.

Learning efficiency and decision-making were assessed in the T-maze by analyzing the time spent in each zone, reflecting the zebrafish’s ability to retain spatial cues and navigate confidently toward the green (left) zone. As shown in Fig. 8a, BPA at 4 mg/L resulted in a 26.8 % increase in time spent in the home zone compared to control (F(4,20) = 8.905, p = 0.0003, η² = 0.6404; mean difference = 30.70, 95 % CI: −60.22 to −1.18, p = 0.0390), suggesting preference for a familiar space, possibly due to anxiety-driven avoidance behavior. Similarly, AlCl₃ at 2 mg/L showed a 21.5 % increase (mean difference = 27.04, 95 % CI: −42.78–86.58, p = 0.4475), indicating hesitancy to explore reward-associated zones. Time spent in the green (left) zone was significantly reduced in the BPA 4 mg/L group (F(4,20) = 10.78, p < 0.0001, η² = 0.6881; 39.2 %, mean difference = 38.86, 95 % CI: 18.50–58.82, p = 0.0001) and AlCl₃ 2 mg/L group (mean difference = 27.88, 95 % CI: 7.72–48.04, p = 0.0041), indicating impaired memory retention and reduced goal-directed motivation (Fig. 8b). AlCl₃ 4 mg/L also showed a 34.7 % reduction (mean difference = 29.90, 95 % CI: 7.51–47.84, p = 0.0044), further supporting deficits in spatial learning and reward recognition. As shown in Fig. 8c, both BPA 4 mg/L and AlCl₃ 4 mg/L significantly increased time spent in the red (right) zone (F(4,20) = 7.126, p = 0.0010, η² = 0.5877; by 38.3 %, mean difference = 44.08, 95 % CI: −78.48 to −9.17, p = 0.0003, and 35.1 %, mean difference = 30.28, 95 % CI: 0.76–69.80, p = 0.0428, respectively), indicating a behavioral shift away from learned spatial cues and reward zones.

#### 3.3.3 Effect on spatial preference and memory retention

**Fig. 9:**
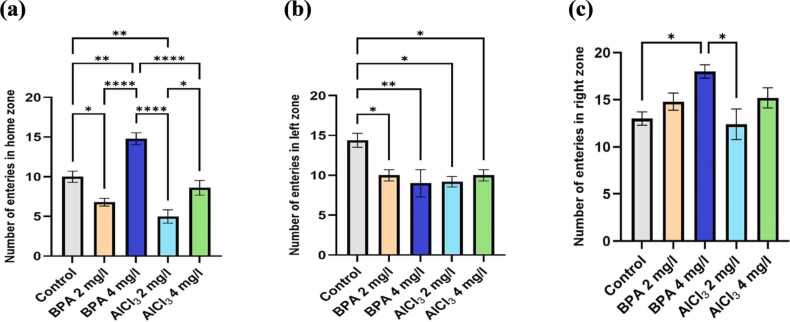
a) Number of entries in home zone, b) Number of entries in left zone or green zone, c) Number of entries in right zone or red zone. The data were expressed as mean ± SEM based on individual zebrafish replicates (n = 7), with each fish undergoing three repeated behavioral trials. Statistical significance was assessed using a one-way ANOVA followed by Tukey's post hoc test. Significance levels were denoted as *p < 0.05, **p < 0.01, ***p < 0.001, and ****p < 0.0001.

Spatial memory and preference were assessed in the T-maze by quantifying the number of entries into each zone, indicating how effectively zebrafish recalled the reward-associated area. As shown in Fig. 9a, BPA at 4 mg/L resulted in a 23.4 % increase in home zone entries (F(4,20) = 4.816, p = 0.0089, η² = 0.4908; mean difference = 3.20, 95 % CI: 0.01–6.39, p = 0.0490), while AlCl₃ at 2 mg/L and 4 mg/L increased entries by 18.6 % and 21.2 %, respectively (mean differences = 3.20, 95 % CI: 0.79–5.62, p = 0.0142 for AlCl₃ 2 mg/L; and 3.80, 95 % CI: 1.13–6.47, p = 0.0081 for AlCl₃ 4 mg/L), compared to control. These findings suggest a tendency to revisit familiar areas rather than actively explore the reward-associated green zone.

As illustrated in Fig. 9b, the BPA 4 mg/L and AlCl₃ 2 mg/L groups exhibited significant reductions in green zone entries (F(4,20) = 24.49, p < 0.0001, η² = 0.8304; mean differences = 5.40, 95 % CI: 1.13–9.67, p = 0.0092 for BPA 4 mg/L; and 5.20, 95 % CI: 0.92–9.48, p = 0.0157 for AlCl₃ 2 mg/L; corresponding to 35.8 % and 28.4 %, respectively), indicating diminished exploratory motivation and impaired spatial learning. AlCl₃ 4 mg/L also showed a 30.6 % decrease (mean difference = 4.80, 95 % CI: 1.13–8.47, p = 0.0166), while BPA 2 mg/L showed a non-significant 12.7 % reduction (mean difference = 1.00, 95 % CI: −1.08–3.08, p = 0.3547), suggesting a potential dose-dependent decline in reward-directed navigation.

In the red (non-reward) zone (Fig. 9c), entry frequency significantly increased in the BPA 4 mg/L (F(4,20) = 4.284, p = 0.0115, η² = 0.4614; mean difference = 4.40, 95 % CI: 0.13–8.87, p = 0.0417) and AlCl₃ 4 mg/L (mean difference = 5.20, 95 % CI: 0.92–9.48, p = 0.0157) groups compared to control, suggesting a shift in spatial preference toward non-rewarded regions. BPA 2 mg/L and AlCl₃ 2 mg/L groups showed non-significant increases of 13.9 % (mean difference = 1.80, 95 % CI: −2.09–5.69, p = 0.3446) and 11.5 % (mean difference = 2.80, 95 % CI: −1.70–7.29, p = 0.3872), respectively, indicating only mild spatial alterations at lower exposures.

### 3.4 Histopathological analysis

**Fig. 10:**
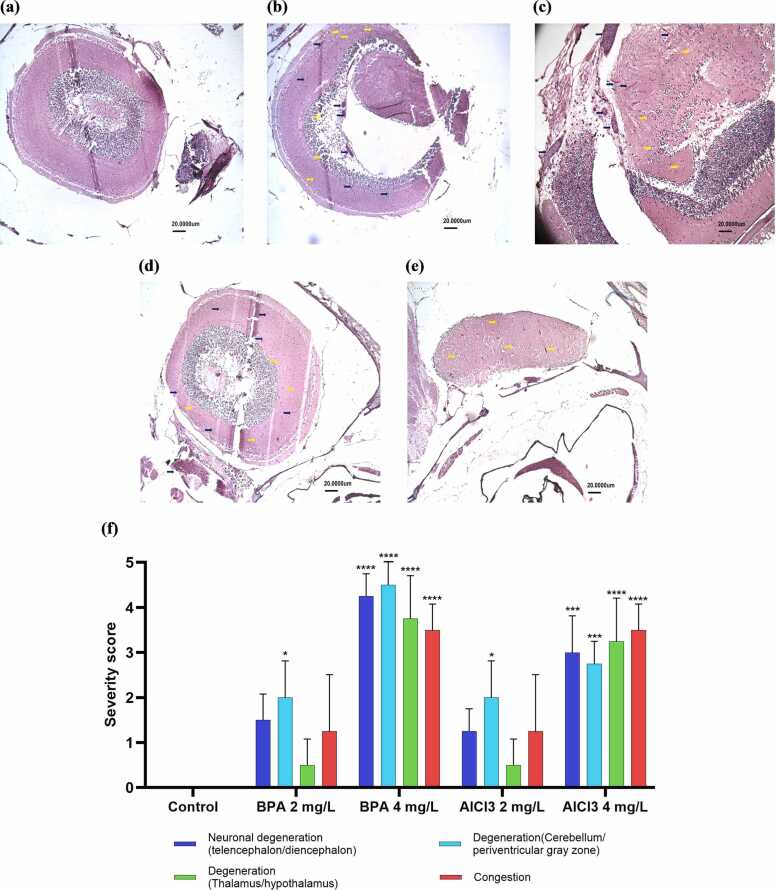
Histopathological alterations in the zebrafish brain following exposure to BPA and AlCl₃ in different experimental groups. (a) Control showing normal histoarchitecture of brain (Teo-optic tectum, PGZ-periventricular grey zone, Tsc-central nucleus of torus semicircularis, TTB-tectobulbular tract, Tla-lateral torus), (b) BPA 2 mg/L, (c) BPA 4 mg/L, (d) AlCl₃ 2 mg/L, and (e) AlCl₃ 4 mg/L, (f) Grouped bar chart showing the semi-quantitative lesion severity scores across different brain regions and treatment groups. Data are expressed as mean ± SEM (n = 3). Statistical analysis was performed using two-way ANOVA followed by Tukey’s multiple comparison test to assess the effects of treatment and brain region. Significance levels are indicated as *p < 0.05, **p < 0.01, ***p < 0.001, and ****p < 0.0001. All histological images were captured at 10 × magnification. Yellow arrows indicate neuronal degeneration, pyknotic neurons, and vacuolation, while blue arrows indicate congestion. Scale bar = 20 µm. Histopathological scoring was performed independently by two blinded pathologists using a 5-point severity scale based on goRENI/INHAND guidelines (n = 3 fish per group).

Histopathological analysis of zebrafish brain tissues across experimental groups demonstrated a dose-dependent neurotoxic impact of BPA and AlCl₃, with progressive neuronal degeneration observed at higher exposure levels. The control group (G1) exhibited intact neuroanatomical structures with no evidence of histopathological alterations (Fig. 10a). In contrast, the BPA 2 mg/L (G2) and AlCl₃ 2 mg/L (G4) groups showed mild neuronal degeneration and vacuolation in the telencephalon, diencephalon, and cerebellum, accompanied by occasional pyknotic neurons and minimal perivascular congestion (Fig. 10b, d). The BPA 4 mg/L group (G3) exhibited more pronounced neurodegenerative changes, including increased neuronal vacuolation, expanded perineural spaces, and marked congestion, particularly in the telencephalon, diencephalon, periventricular gray zone, and cerebellum (Fig. 10c). Similarly, AlCl₃ 4 mg/L (G5) induced mild-to-moderate neuronal degeneration, with notable structural disruptions in the thalamus, cerebellum, and telencephalon, including pyknotic neurons, vacuolation, and prominent vascular congestion (Fig. 10e). The severity of these neurodegenerative changes was greater in the BPA 4 mg/L group compared to AlCl₃ at equivalent concentrations, indicating a higher neurotoxic potential for BPA. These findings are further supported by the grouped bar chart (Fig. 10f), which quantitatively illustrates the incidence and severity of specific histopathological lesions across all experimental groups.

## 4 Discussion

Neurodegenerative disorders such as Alzheimer’s disease (AD) are marked by progressive neuronal loss, leading to cognitive decline, behavioral impairments, and emotional disturbances. While familial AD accounts for a small fraction of cases, approximately 95 % are sporadic, with increasing evidence linking their onset to prolonged exposure to environmental neurotoxicants [24]. Despite the availability of various neurotoxin-induced models, many fall short of replicating the chronic, multifactorial progression observed in human AD. Aβ-based models simulate amyloid deposition but lack tau pathology and sustained oxidative stress [25]. Okadaic acid induces tau hyperphosphorylation but does not model amyloid accumulation and exhibits high cytotoxicity [26]. Scopolamine causes transient cholinergic dysfunction without structural degeneration [27], while STZ models metabolic deficits with limited amyloid or tau relevance [28]. Similarly, LPS, MPTP, and rotenone models primarily target dopaminergic systems or acute inflammation, aligning more with Parkinsonian syndromes [29], [30], [31]. D-galactose models emphasize oxidative aging but fail to replicate core AD features such as amyloid plaques and tau tangles [32].

BPA and AlCl₃ were selected due to their widespread use, human exposure risk, and reported links to neurodegenerative outcomes. BPA is among the most produced industrial chemicals, present in polycarbonates, canned food linings, and thermal papers, with expected global production to exceed 10 million tons by 2030. Despite regulations, post-COVID increases in plastic packaging and food handling have led to elevated human BPA exposure [33]. AlCl₃ is used in cookware, food additives, and water treatment, and is known to cross the blood-brain barrier, inducing tau hyperphosphorylation and cholinergic deficits [34]. Chronic exposure to both chemicals has been associated with cognitive dysfunction, reduced IQ, and impaired learning across various age groups. Unlike other neurotoxicants that target isolated pathways or lack real-world exposure relevance, BPA and AlCl₃ were specifically chosen for their dual significance: BPA primarily disrupts synaptic and hormonal regulation, while AlCl₃ is mechanistically linked to tauopathy and structural neurodegeneration, offering a complementary perspective on environmental neurotoxicity [35]. The continued industrial use beyond regulatory thresholds, especially in low- and middle-income countries, underscores the relevance of this comparative study. The exclusion of female zebrafish in this study was intentional to minimize hormonal fluctuations that could confound neurobehavioral readouts, particularly those related to anxiety, cognition, and spatial exploration. Future studies will incorporate sex-based comparisons to uncover potential sex-specific vulnerabilities to BPA and AlCl₃ exposure.

Although the neurotoxic potential of BPA has been widely investigated, existing zebrafish-based studies exhibit several critical limitations that constrain their translational relevance. A predominant issue is the reliance on acute or short-term exposure durations (typically 24–96 h), which fail to capture the effects of chronic, cumulative environmental exposure [12]. Most studies, such as those evaluating BPAnalogues like BPA, BPF, and BPAF, have focused on embryonic stages and social behavior endpoints, whereas our study overcomes this by employing adult zebrafish, assessing chronic exposure, and incorporating multi-dimensional behavioral and histopathological analyses relevant to neurodegenerative outcomes [36]. Additionally, most prior studies focus on larval zebrafish, which, while advantageous for high-throughput screening, lack the behavioral complexity observed in adults. This restricts assessments of higher-order functions such as cognition, memory, and anxiety, which were robustly addressed in the present study through NTDT and color-based appetite T-maze assays [37]. Many BPA studies also utilize supra-environmental concentrations (>10 mg/L), limiting ecological relevance and obscuring low-dose effects. By contrast, our study adopted moderate, environmentally aligned doses (2 and 4 mg/L) and demonstrated clear, dose-dependent behavioral and histopathological changes [38]. Moreover, previous research often limits behavioral endpoints to general locomotion or hypoactivity, omitting key parameters like spatial preference, latency to top zone, and zone crossings. These were comprehensively analysed here, offering a multidimensional view of BPA-induced anxiety and exploratory suppression [39]. Another common limitation is the scarcity of histopathological validation to support behavioral outcomes. The current study addressed this gap by incorporating detailed brain histology, confirming region-specific neuronal damage, particularly in the telencephalon and diencephalon [40]. Furthermore, while recent BPA research increasingly explores the efficacy of protective agents (e.g., quercetin, taurine, biochanin A, Ulva lactuca), the toxicant's independent effects are often under-characterized. Unlike those intervention-focused studies, our model provides a clear, mechanistic profile of BPA-induced neurotoxicity without confounding therapeutic variables [12], [41], [42], [43].

Existing studies utilizing AlCl₃ to model neurotoxicity in zebrafish often exhibit significant limitations, particularly concerning dose selection and exposure duration. Many have relied on larval or embryonic models and used concentrations near or exceeding the LC₅₀ (36.5–59.1 mg/L for larvae over 96 h), leading to systemic toxicity, developmental anomalies, and mortality—conditions unsuitable for behavioral or histopathological evaluations. Furthermore, high-dose, short-duration models (4–10 days) often produce non-specific toxic effects like stress and organ failure, confounding interpretations related to neurodegeneration. Compared to rodent studies, AlCl₃ zebrafish models remain scarce, with inconsistent dosing protocols, minimal behavioral endpoints, and limited histological validation, hindering a clear understanding of its dose-dependent neurotoxicity in aquatic systems [19], [44].

To address these gaps, the present pilot study adopted a 21-day chronic exposure protocol using environmentally relevant, sublethal doses (2 and 4 mg/L) of both BPA and AlCl₃ in adult zebrafish. This design ensured zero mortality and absence of systemic toxicity, enabling accurate assessment of behavioral impairments and region-specific neuronal damage. The model integrates locomotor, cognitive, and anxiety-related behavioral assays with histopathological correlation, offering a comprehensive and translational framework for environmental neurotoxicity screening. Notably, this is the first study to directly compare the neurotoxic profiles of BPA and AlCl₃ under identical, dose-matched conditions, providing novel insights into their relative neurotoxic potency and addressing a pressing need for comparative environmental risk assessment.

The NTDT is a widely accepted behavioral assay in zebrafish research, used to assess both anxiety-like behavior and general locomotor activity, analogous to open-field and elevated-plus maze tests in rodents [45]. In the present study, zebrafish exposed to BPA and AlCl₃ exhibited a clear, dose-dependent decline in locomotor performance, with the BPA 4 mg/L group showing the most pronounced reduction - 36.7 % decrease in total distance travelled and reduced swimming speed (see Fig. 2a–c, Fig. 3c). These outcomes suggest hypoactivity and neuromotor dysfunction indicative of early-stage neurotoxicity. This is consistent with previous findings reporting locomotor suppression and oxidative stress-associated neuronal degeneration following BPA exposure in zebrafish [46]. Moreover, the reduced line crossings and delayed top-zone entries observed in BPA-treated fish (Fig. 2c, Fig. 5) indicate impaired sensorimotor coordination and motivational decline. These findings are supported by earlier studies that reported diminished zone transitions and reduced top-zone exploration, reinforcing the impact of BPA on exploratory behavior [12]. Previous studies have also reported increased bottom-dwelling, delayed vertical movement, reduced swimming velocity, and impaired habituation in BPA-exposed zebrafish, supporting the anxiogenic profile observed [12], [40]. Mechanistically, these behaviors are consistent with previous reports linking BPA exposure to disruptions in calcium signaling and synaptic plasticity, alongside serotonergic and oxidative imbalances [4], [47]. Additionally, BPA's endocrine-disrupting nature may also influence the hypothalamic–pituitary–interrenal (HPI) axis, a pathway analogous to mammalian HPA-which could contribute to anxiety-like responses. In contrast, AlCl₃ exposure produced a milder phenotype. Although AlCl₃ 4 mg/L caused a 19.6 % reduction in swim speed and altered spatial preference (Figs. 2b, 3e), its impact was notably less severe. Similar reductions in exploratory drive have been observed, attributed to aluminum-induced mitochondrial dysfunction and localized synaptic impairment [19], [44]. Together, these findings demonstrate that both compounds impair locomotor and anxiety-related behavior, though BPA induces more pronounced and systemic disruption.

The color-based appetite T-maze test provided insight into spatial learning, reward memory, and cognitive flexibility. BPA exposure, particularly at 4 mg/L, led to a 35.8 % reduction in reward-zone entries, increased non-rewarded zone preference, and lower exploratory transitions (Fig. 6a–c, Fig. 7c). These outcomes are consistent with findings that BPA exposure impairs spatial memory and induces learning and behavioral deficits in zebrafish [48]. Reduced task initiation and prolonged home-arm dwelling in BPA-treated groups (Fig. 8b–c) may indicate motivational decline and executive dysfunction, possibly driven by dopaminergic and glutamatergic dysregulation. Additionally, BPA-induced neuroinflammation has been linked to gut-brain axis disruption, offering a systemic perspective on cognitive inflexibility [49]. In comparison, AlCl₃ caused moderate cognitive deficits, including a 30.6 % reduction in rewarded entries (Fig. 8 and Fig. 9), but exploratory activity remained relatively intact. AlCl₃ at 2 mg/L and 4 mg/L led to 18.6 % and 21.2 % increases in home-zone entries, respectively, and a 28.4 % and 30.6 % reduction in green (reward) zone entries, indicating reduced spatial learning and memory retention. Moreover, time spent in the red (non-reward) zone increased by 35.1 % in the AlCl₃ 4 mg/L group, reflecting a shift in spatial preference and impaired decision-making. Such gradual impairment aligns with earlier findings associating AlCl₃ exposure with cholinergic disruption, tauopathy-related toxicity [50], while further studies have shown that aluminum impairs spatial memory via mitochondrial dysfunction and localized synaptic impairment [19]. Thus, while both agents impair cognition, BPA exerts broader, faster-acting effects on behavioral flexibility, whereas AlCl₃ induces subtler but persistent deficits through progressive neurochemical and structural disturbances.

Histological findings corroborated behavioral outcomes, revealing widespread neurodegeneration in BPA-treated zebrafish. In the 4 mg/L group, cytoplasmic vacuolization, neuronal pyknosis, and perineural edema were prominent in the telencephalon, diencephalon, and cerebellum—regions vital to cognitive and motor integration (Fig. 10 c). These results are consistent with prior studies reporting BPA-induced neurodegeneration, neuronal apoptosis, and oxidative stress in zebrafish brain regions [21], [46]. The elevated histological score (mean = 4.0) reflects the compound’s high neurotoxic potential. In contrast, AlCl₃ induced moderate degeneration, mainly in the thalamus and cerebellum, with milder vacuolization and glial swelling (Fig. 10e). The average histological score for AlCl₃ 4 mg/L was lower (mean = 3.13), indicating a comparatively milder degree of neurodegeneration than BPA. These findings align with studies highlighting Aluminum's selective action in cholinergic-rich regions and region-specific oxidative stress [51]. Mitochondrial compromise and gliosis have also been reported without widespread cell loss [50]. These differential effects are consistent with the possibility of distinct mechanistic pathways. BPA may influence synaptic plasticity and HPI axis signaling, whereas AlCl₃ could involve mitochondrial dysfunction and tauopathy, as suggested by previous reports [52], [53], [54], [55]. However, these hypotheses require confirmation through targeted molecular analyses.

This study presents a comprehensive comparative model for assessing environmental neurotoxicants using adult zebrafish. The findings demonstrate that BPA is more potent than AlCl₃ in disrupting neurobehavioral and histological parameters. Beyond implications for aquatic toxicology, this model may also inform evaluations of potential human health risks, particularly in regions with high plastic or aluminum contamination via industrial or agricultural runoff. Future work should explore molecular pathways, sex-based differences, long-term impacts, and potential neuroprotective strategies such as antioxidants or therapeutic agents to mitigate the effects of these pervasive contaminants.

## 5 Conclusion

This study establishes that chronic exposure to BPA elicits more severe neurobehavioral impairments and neuropathological alterations than AlCl₃ in adult zebrafish, possibly through multifactorial mechanisms involving endocrine disruption, oxidative stress, and synaptic dysfunction. By integrating locomotor, cognitive, and anxiety-related behavioral assays with detailed brain histopathology, the findings underscore BPA’s broader and more systemic neurotoxic potential, whereas AlCl₃ produced moderate, region-specific effects, primarily through mitochondrial and cholinergic pathways. These results validate adult zebrafish as a sensitive and translational model for environmental neurotoxicity and highlight the potential ecological and human health risks associated with chronic, low-dose exposure to these widespread aquatic contaminants. Future studies should incorporate molecular biomarker analyses, sex-based comparisons, and environmentally realistic mixture exposures to enhance mechanistic insight and regulatory relevance.

## 6 Limitations

Although this study provides robust behavioral and histopathological evidence on the comparative neurotoxicity of BPA and AlCl₃, several limitations warrant consideration. The use of only adult male zebrafish precludes assessment of sex- and developmental-stage-specific vulnerabilities, which are relevant for endocrine disruptors like BPA. This choice minimized variability caused by hormonal fluctuations in females, which could confound neurobehavioral outcomes. While freezing episodes and erratic swimming are recognized anxiety indicators, these were not included here and will be added in future work to strengthen behavioral profiling. The absence of molecular-level validations such as oxidative stress biomarkers, neurotransmitter profiling, or apoptotic gene expression limits mechanistic depth. Statistical comparisons used one-way ANOVA; future studies with larger cohorts should employ two-way ANOVA to better capture main and interaction effects. Addressing these gaps in future research will enhance translational and ecological relevance.

## CRediT authorship contribution statement

**B Logeshwari:** Writing – review & editing, Writing – original draft, Visualization, Validation, Software, Resources, Project administration, Methodology, Investigation, Formal analysis, Data curation, Conceptualization. **Gayathri Veeraraghavan:** Writing – review & editing, Visualization, Supervision, Resources, Project administration, Conceptualization. **Srikanth Jeyabalan:** Writing – review & editing, Validation, Supervision, Software, Resources, Project administration, Methodology, Investigation, Formal analysis, Conceptualization. **K Krishnaraj:** Writing – review & editing, Validation, Supervision, Methodology, Conceptualization. **Chetan Ashok:** Writing – review & editing, Visualization, Validation, Resources, Methodology. **Naveen Kumar Rajasekaran:** Methodology, Investigation, Formal analysis. **Mahendran Sekar:** Supervision, Formal analysis, Conceptualization. **Ling Shing Wong:** Writing – review & editing, Supervision, Investigation, Conceptualization. **Vetriselvan Subramaniyan:** Writing – review & editing, Visualization, Supervision, Formal analysis, Conceptualization.

## Ethics approval and consent to participate

The present study was approved by the Institutional Animal Ethical Committee (IAEC) of Sri Ramachandra Institute of Higher Education and Research (Deemed to be University), Chennai, India (Approval No. IAEC/71/SRIHER/863/2023; Chennai, India). The study was conducted in accordance with the guidelines of the CCSEA, and adhered to the principles of the 3Rs to ensure ethical and responsible use of animals in research. All experimental procedures involving zebrafish were designed and reported in compliance with CCSEA regulations and the ARRIVE 2.0 guidelines to ensure scientific rigor, reproducibility, and transparency in animal research.

## Funding

This study was supported by the Founder-Chancellor Shri N.P.V. Ramasamy Udayar Fellowship from Sri Ramachandra Institute of Higher Education and Research (SRIHER), Chennai and and INTI International Research Fellowship Program (IIU/HR/JL/NHZ/12566/ 23), INTI University, Malaysia for the year 2023-2025.

## Declaration of Competing Interest

The authors declare the following financial interests/personal relationships which may be considered as potential competing interests: The authors declare that there are no other relationships or activities that could be perceived to have influenced the submitted work. If there are other authors, they declare that they have no known competing financial interests or personal relationships that could have appeared to influence the work reported in this paper.

## Data Availability

Data will be made available on request.
